# Body mass index influences the response to infliximab in ankylosing spondylitis

**DOI:** 10.1186/ar3841

**Published:** 2012-05-14

**Authors:** Sébastien Ottaviani, Yannick Allanore, Florence Tubach, Marine Forien, Anaïs Gardette, Blandine Pasquet, Elisabeth Palazzo, Marine Meunier, Gilles Hayem, Chantal Job-Deslandre, André Kahan, Olivier Meyer, Philippe Dieudé

**Affiliations:** 1 Rheumatology Department, AP-HP, Paris Diderot, Sorbonne Paris Cité University, Bichat Claude Bernard Hospital, 46 rue Henri Huchard, Paris, 75018, France; 2 Rheumatology A Department, AP-HP, Paris Descartes University, Cochin Hospital, 27 rue du Faubourg Saint-Jacques, Paris, 75014, France; 3 INSERM U1016, Paris Descartes University, Cochin Hospital, 27 rue du Faubourg Saint-Jacques, Paris, 75014, France; 4 Epidemiology Biostatistics and Clinical Research Department, AP-HP, INSERM, CIE801, Paris Diderot, Sorbonne Paris Cité University, Bichat Claude Bernard Hospital, 46 rue Henri Huchard, Paris, 75018, France; 5 INSERM U699, Paris Diderot, Sorbonne Paris Cité University, Faculté de Médecine Xavier Bichat, 16 rue Henri Huchard, Paris, 75018, France

## Abstract

**Introduction:**

The excess of adipose tissue in obese individuals may have immunomodulating properties and pharmacokinetic consequences. The aim of this study was to determine whether body mass index (BMI) affects response to infliximab (IFX) in ankylosing spondylitis (AS) patients.

**Methods:**

In 155 patients retrospectively included with active AS, the BMI was calculated before initiation of IFX treatment (5 mg/kg intravenously). After 6 months of treatment, changes from baseline in BASDAI, Visual Analogue Scale (VAS) pain, C-reactive protein (CRP) level, and total dose of nonsteroidal antiinflammatory drug (NSAID) were dichotomized with a threshold corresponding to a decrease of 50% of initial level of the measure, into binary variables assessing response to IFX (BASDAI50, VAS50, CRP50, NSAID50). Whether the BMI was predictive of the response to IFX therapy according to these definitions was assessed with logistic regression.

**Results:**

Multivariate analysis found that a higher BMI was associated with a lower response for BASDAI50 (*P *= 0.0003; OR, 0.87; 95% CI (0.81 to 0.94)), VAS50 (*P *< 0.0001; OR, 0.87; 95% CI (0.80 to 0.93)); CRP50 (*P *= 0.0279; OR, 0.93; 95% CI (0.88 to 0.99)), and NSAID50 (*P *= 0.0077; OR, 0.91; 95% CI (0.85 to 0.97)), criteria. According to the three WHO BMI categories, similar results were found for BASDAI50 (77.6%, 48.9%, and 26.5%; *P *< 0.0001), VAS50 (72.6%, 40.4%, and 16.7%; *P *< 0.0001); CRP50 (87.5%, 65.7%, and 38.5%; *P *= 0.0001), and NSAID50 (63.2%, 51.5%, and 34.6%; *P *= 0.06).

**Conclusions:**

This study provides the first evidence that a high BMI negatively influences the response to IFX in AS. Further prospective studies, including assessment of the fat mass, pharmacokinetics, and adipokines dosages are mandatory to elucidate the role of obesity in AS IFX response.

## Introduction

Overweight and obesity are defined as abnormal or excessive fat accumulation that presents a risk to health [1]. A crude population measure of obesity is the body mass index (BMI). Individuals with a BMI of 30 kg/m^2 ^or more are considered obese.

It is now well known that pharmacokinetic variables of drug clearance and volume of distribution (V_d_) could be influenced by overweight and obesity [2]. In addition, adipose tissue can exert both endocrine and immune effects on multiple other organs through the release of adipocytokines [3], which are suspected to contribute to the pathogenesis of several inflammatory conditions, including rheumatoid arthritis (RA) [4]. Even if to date and to our knowledge, the role of fat tissue has not been widely investigated in ankylosing spondylitis (AS), several indirect results suggest a possible link between the AS-related inflammation and fat tissue excess: (a) weight loss and subsequent lower BMI are linked to a high RA activity [5]; and (b) a significant increase in body weight and fat mass has been observed in AS patients receiving anti-tumor necrosis factor (TNF)-α treatment [6].

In addition to its potential implication in the inflammatory process of rheumatic conditions, a recent report has suggested that fat mass may also affect the response to therapy by showing a negative correlation between BMI and response to infliximab (IFX) in RA [7]. Indeed, the authors observed that RA patients with a high BMI responded less well to IFX, a finding that held true when adjusted for the baseline DAS28 or anti-citrullinated protein antibody status [7]. Hence, those results led to the hypothesis that BMI could be considered a new predictive marker of IFX response in inflammatory diseases, including at least RA. With regard to the critical issue that remains, the identification of predictors to biologics, we aimed to investigate whether the BMI could influence the IFX response in AS.

## Materials and methods

### Study population

We performed a retrospective study including 155 consecutive individuals fulfilling the European Spondyloarthropathy Study Group (ESSG) AS criteria [8], who have received or receive infliximab. All patients had active AS, according to the Assessment of SpondyloArthritis Society (ASAS) criteria [9]. Participating centers were the Rheumatology A Department of Cochin hospital and the Rheumatology Department of Bichat Hospital, Paris, France. The following data were collected at baseline (M0) and at month 6 (M6) for the analysis: BMI, gender, age, disease duration, Bath Ankylosing Spondylitis Disease Activity Index (BASDAI), pain visual analogue scale (VAS), use of nonsteroidal antiinflammatory drugs (NSAIDs), *HLA B27 *status, and C-reactive protein (CRP) level. BMI was calculated by weight in kilograms divided by height in square meters at baseline and M6. According to the WHO criteria, normal BMI was defined as a BMI < 25 kg/m^2^; overweight, as a BMI of 25 to 30 kg/m^2^; and obesity, as a BMI > 30 kg/m^2 ^[1]. The baseline demographic and clinical features of the AS patients are summarized in Table 1. Infliximab was given intravenously at 5 mg/kg every 6 weeks, according to international recommendations [9].

**Table 1:** Baseline characteristics of 155 AS patients according to the three WHO BMI categories

	Whole AS population (*n *= 155)	BMI < 25 kg/m^2^ (*n *= 63)	BMI [25-30] kg/m^2^ (*n *= 54)	BMI > 30 kg/m^2^ (*n *= 38)	*P *value
Age (years), median [IQR]	43.1 [35.0-51.8]	38.5 [31.6-48.6]	45.0 [35.6-52.6]	45.4 [40.0-50.0]	0.0127
Male gender, *n *(%)	98 (63.3)	45 (71.4)	36 (66.6)	17 (44.7)	0.0214
*HLAB27*, *n *(%)	96 (64.9)	48 (76.2)	28 (57.1)	20 (55.6)	0.0451
Disease duration (years), median [IQR]	8.0 [3.0-12.0]	8.0 [4.0-12.0]	7.0 [3.0-11.0]	6.0 [3.0-12.0]	0.7640
BASDAI (0 to -100 mm), median [IQR]	60.0 [47.5-70.0]	60.0 [50.0-73.0]	56.0 [48.0-66.0]	62.0 [34.0-70.0]	0.4782
VAS pain (0 to 100 mm), median [IQR]	61.0 [50.0-75.0]	65.0 [50.0-80.0]	60.0 [50.0-70.0]	70.0 [49.0-75.0]	0.4598
Use of NSAIDs (% of maximal dose), mean (SD)	62.2 (45.7)	72.2 (42.9)	53.7 (42.9)	57.4 (46.0)	0.0703
CRP (mg/dl), median [IQR]	10.0 [5.0-24.0]	11.0 [5.0-23.0]	14.0 [5.0-33.0]	7.9 [5.0-16.0]	0.2550

AS, Ankylosing spondylitis; BASDAI; Bath ankylosing spondylitis disease activity index; BMI, body mass index; CRP, C-reactive protein; IQR, interquartile range NSAID, nonsteroidal antiinflammatory drugs; VAS, visual analogue scale.

Clinical response was assessed after 6 months of IFX therapy. For each of these criteria, change from baseline was dichotomized, with a threshold corresponding to a decrease of 50% of the initial level of the measure, into binary variables corresponding to different definitions of response to IFX (that is, BASDAI50, VAS50, CRP50, and NSAID50). The BASDAI20 and BASDAI70 also were assessed. No specific recommendation was made regarding the use of NSAIDs according to the IFX response.

The study was approved by the local institutional review board (Number 11-089), and written informed consent was obtained from all subjects in the study.

### Statistical analysis

Continuous variables are expressed as mean (SD) or median (IQR). Categoric variables are expressed as frequencies and percentages. Comparisons between categoric variables were performed by using the Pearson χ^2 ^test. The Student *t *test (two-tailed) was used to compare normally distributed continuous variables, and the Wilcoxon rank-sum test for continuous variables not normally distributed.

To determine predictors of the response to IFX according to the different definitions of this response (BASDAI20, BASDAI50, BASDAI70, VAS50, CRP50, and NSAID50), we fitted logistic regressions. We included in the first models all variables significantly associated with each dependant variable in univariate analyses to a *P *level of 0.20. BMI was considered as a continuous variable in the multivariate analyses.

Several methods were implemented to select the variables to include in the final models (forward, backward, and stepwise). Indexes of goodness-to-fit were calculated, as the AUC and Hosmer and Lemeshow test. The AIC index also was calculated at each step.

To investigate further whether the BMI influenced the BASDAI response criteria 6 months after initiation of IFX therapy, we plotted each of the BASDAI response criteria according to BMI considered in three levels (< 25 kg/m^2^; 25 to 30 kg/m^2^; and > 30 kg/m^2^).

Statistical analysis was performed by using SAS software version 9.2 (SAS Institute Inc., Cary, NC, USA).

## Results

### Characteristics of ankylosing spondylitis patients at baseline

During the 6-month period reported here, two patients stopped the IFX therapy: one for lack of response, and one for infectious pneumonitis. At month 6, among the 155 AS patients consecutively included, the BASDAI response was not available for 14 of them.

In accordance with the WHO definition [1], distribution of individuals having normal weight, overweight, and obesity was 41%, 35%, and 24%, respectively. Characteristics of the three BMI populations are summarized in Table 1. Age (*P *= 0.0127), male gender (*P *= 0.0214), and *HLAB27*-positive status (*P *= 0.0451) at baseline were significantly different across the three BMI groups. Other characteristics showed no significant differences (Table 1). No difference was noted for the characteristics of AS patients between the two participating centers (data not shown)

For all multivariate models, all methods implemented to select variables to include in the final models (forward, backward, and both) proposed the same final models.

### BMI is associated with a lower rate of response to infliximab in ankylosing spondylitis after 6 months of therapy, according to the BASDAI

After 6 months of IFX therapy, 55.4% of AS patients achieved the BASDAI50 response. Univariate analysis found an association between several factors and nonresponse to IFX at M6: BMI (*P *< 0.0001), female gender (*P *= 0.0102), and age at inclusion (*P *= 0.0469) (Table 2). Conversely, BASDAI50 responders were found to have a high level of CRP compared with nonresponders (*P *= 0.0039). Additionally, the *HLAB27-*positive status was found to be more frequent in the BASDAI50-responder subgroup (*P *= 0.047; Table 2).

**Table 2:** Baseline characteristics of the ankylosing spondylitis patients treated with infliximab according to the response BASDAI50 after 6 months of infliximab

	Yes (*n *= 77)^a^	No (*n *= 62)^a ^	*P *value
Age (years), median (IQR)	39.4 (32.0 -50.0)	44.8 (38.2-53.0)	0.0469
Gender (% males)	72.7	51.6	0.0102
Disease duration (years), median (IQR)	8.0 (4.0-14.0)	6.0 (3.0-12.0)	0.1966
BMI (kg/m^2^)	24.4 (21.9-26.8)	28.7 (25.6-31.6)	< 0.0001
*HLAB27 *(% of patients)	73.3	56.9	0.0470
Δweight (kg), median (IQR)	-1.0 (-2.0-2.0)	0.0 (-2.0-3.0)	0.4948
Baseline CRP (mg/dl), median (IQR)	14.0 (5.0-26.0)	6.6 (3.0-13.0)	0.0039

**^a^**Two patients withdrew from IFX therapy (lack of response and serious adverse event) and BASDAI50 lacking data for 14 AS patients

BASDAI50, 50% improvement in the Bath Ankylosing Spondylitis Disease Activity Index; BMI, body mass index; CRP, C-reactive protein; IQR, interquartile range.

**Figure 1:**
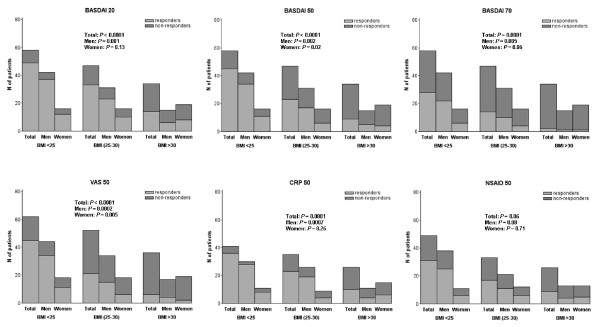
**Clinical response and body mass index (BMI) in ankylosing spondylitis (AS) patients after 6 months of treatment with infliximab, according to the three WHO BMI categories**. BASDAI, Bath Ankylosing Spondylitis Disease Activity Index; CRP, C-reactive protein; NSAIDs, nonsteroidal antiinflammatory drugs; VAS, visual analogue scale.

We next investigated whether the BMI at inclusion, according to the three WHO BMI categories, could influence the BASDAI response after 6 months of IFX therapy. We observed a strong decrease of the proportion of responders in normal to overweight and obese AS patients: 77.6%, 48.9%, and 26.5%, respectively (*P *< 0.0001) (Figure 1).

Interestingly, a significant linear decrease of frequency of BASDAI20 responders according to the three BMI categories also was observed: 84.5%, 70.2%, and 41.2%, respectively (*P *< 0.0001). When the BASDAI70 was queried, similar results were observed: 48.3%, 29.8%, and 5.9% (*P *< 0.0001), respectively (Figure 1). When analysis was dichotomized according to the gender, a significant decrease of BASDAI20 (*P *= 0.0011), BASDAI50 (*P *= 0.002), and BASDAI70 (*P *= 0.0055) responses were observed only in male patients.

Multivariate analysis, including gender, identified both baseline BMI and CRP level as independent factors predictive of the BASDAI50 response at month 6: *P *= 0.0003; OR, 0.87; 95% CI (0.81 to 0.94); and *P *= 0.0448; OR, 1.02; and 95% CI (1.00 to 1.04), respectively. Linear regression analysis found a strong correlation between the ΔBASDAI and BMI (*P <*0.0001; *β *= 1.30).

### BMI is associated with a lower rate of response to infliximab in ankylosing spondylitis after 6 months of therapy, according to VAS50, CRP50, and NSAID50

When analysis was performed according to the three BMI categories, we observed a decrease in the proportion of VAS50 responders: 72.6%, 40.4%, and 16.7%, respectively (*P *< 0.0001) (Figure 1). The proportion of CRP50 responders was 87.5% in normal weight, 65.7% in overweight, and 38.5% in obese AS patients (*P *= 0.0001) (Figure 1). In good agreement with the distribution of both VAS50 and CRP50 responses according to the three BMI categories, a linear decrease of proportion of NSAID50 responders also was observed, but not reaching statistical significance: 63.2%, 51.5%, and 34.6%, respectively (*P *= 0.06) (Figure 1). According to the gender, a significant decrease of VAS50 (*P *= 0.0002) and CRP50 (*P *= 0.0007) responses was detected in male patients. In female AS patients, only the VAS50 response was decreased, reaching statistical significance (*P *= 0.005).

Multivariate analysis identified a higher BMI and male gender as independent factors associated with the VAS50 response: OR, 0.87; 95% CI (0.80 to 0.93); *P *< 0.0001; and OR, 2.11; 95% CI (1.01 to 4.41); *P *= 0.0461, respectively (Table 3).

**Table 3:** Response criteria influenced by BMI identified through multivariate analysis: a higher BMI is independently associated with a nonresponse of ankylosing spondyloarthritis to infliximab at month 6^a^

Response criteria	*P *value	OR (95% CI]^a^
BASDAI50	0.0003	0.87 (0.81-0.94)
VAS50	< 0.0001	0.87 (0.80-0.93)
CRP50	0.0279	0.93 (0.88-0.99)
NSAID50	0.0077	0.91 (0.85-0.97)

**^a^**Adjusted on significant factors in univariate analysis (*P *< 0.20). BASDAI, Bath ankylosing spondylitis disease activity index; CRP, C-reactive protein; NSAIDs, nonsteroidal antiinflammatory drugs; VAS, visual analogue scale.

Regarding the CRP50 response, multivariate analysis found a higher BMI and high CRP level at baseline as predictive of the CRP50 response: OR, 0.93; 95% CI (0.88 to 0.99); *P *= 0.0279; and OR, 1.03; 95% CI (1.01 to 1.05); *P *= 0.0022, respectively (Table 3). A higher BMI was found to be negatively associated with the NSAID50 response: OR, 0.91; 95% CI (0.85 to 0.97); *P *= 0.0077 (Table 3).

## Discussion

The excess of adipose tissue in obese individuals may have immunomodulating properties and pharmacokinetics consequences [2]. In this study, we investigated retrospectively whether the BMI could influence the response to IFX in AS patients. Of most interest, although the baseline BASDAI was found to be similar in the three BMI categories of AS individuals (that is, normal, overweight, and obese), multivariate analysis, including gender, identified the BMI as an independent risk factor for a poor response at M6, whatever the response criteria considered (BASDAI50, VAS50, CRP50, or NSAID50). In good agreement with this finding, when BASDAI20, 50, and 70 responses were queried according to the three WHO BMI categories, a higher BMI resulted in a decrease of clinical response to IFX in the whole AS population (Figure 1). However, when analyses were performed according to gender, similar results were observed in both male and female populations, reaching statistical significance only in AS male patients. Nevertheless, the multivariate analysis, including gender as covariate, provides evidence for an independent role of BMI in the IFX response, ruling out a residual confounding by gender.

Both lower BMI and high CRP level at baseline were found to be associated with a good response to IFX. Interestingly, a recent study reported that a high CRP level was associated with a good outcome in AS [10,11]. Nonetheless, in these studies, the BMI was not assessed [10,11]. In addition, and to strengthen the independent role of BMI as a predictive marker of the IFX response, it is of note that, in our study, baseline CRP was not different among the three BMI categories (Table 1).

To our knowledge, this is the first reported study investigating the influence of BMI on the response to IFX in AS. Of most interest, similar results were observed in RA patients treated with IFX, as a significant association between a low BMI and the decrease in the DAS28 after 16 weeks was observed [7]. Additionally, it is now well established that obesity is associated with psoriasis [12]. Interestingly, a previous cohort study reported a decrease of response to systemic agents in psoriasis [13]. A negative impact of weight was also found with ustekinumab and etanercept [14]. However, other studies revealed that BMI did not influence the response to IFX in psoriasis [14,15]. If IFX therapy was found to be associated with a gain of weight, in Crohn disease (CD), to date, no study designed to investigate the role of BMI in modulating the response to IFX is available [16,17]. Most interestingly, a recent study found that CD patients with a low baseline BMI (< 18.5) and those with small-bowel involvement achieved a higher increase in BMI as compared with patients with BMI ≥18.5 or patients without small-bowel involvement [17]. These findings suggest that fat-tissue excess could play a role in modulating the response to anti-TNF-α in RA, AS, psoriasis, and CD.

The rate of response in our study was found to be 49.6% for BASDAI50, which is in line with previous reports [18,19]. Of the most interest, 77.6% of AS patients who were of normal weight achieved the BASDAI50 response compared with 26.5% of obese patients. Hence, obesity should be considered a predictor factor of low-rate response to IFX in AS, leading to a threefold decrease of the response rate.

Although IFX had a dose fixed according to the patient's weight, huge variations of interindividual serum concentration have been widely reported in different inflammatory diseases [20,21]. Unlike that observed in RA [20], treatment failure was not associated with a low circulation concentration of IFX in AS [21]. Further pharmacokinetic findings could explain the negative influence of a high BMI on the IFX response. The volume of distribution, V_d_, of a drug provides an estimate of the extent to which a drug is distributed into extravascular tissues. A wide variation exists in the effect on the V_d_, because the affinity of each drug for the excess adipose tissue is unique. Hence, a nonlipophilic drug, such as IFX, whose distribution into the excess adipose tissue is limited, could alter the V_d _[2]. In addition, tissue blood flow influences drug distribution. Tissue perfusion and cardiac function may be reduced in obese individuals, leading to a decrease of IFX distribution [22].

A direct role of the fat tissue excess through the release of adipocytokines may also contribute to predicting drug response. Nonetheless, the putative influence of IFX therapy on adipocytokines production in AS remains unclear. IFX therapy was reported to increase weight and fat mass in the first 6 months of treatment in AS patients [6]. Conversely, another study failed to detect any association between IFX therapy and leptin serum-level variation [23]. Taking into account that (a) TNF-α induces cachexia and (b) a lower weight is correlated with active RA [5], it could be hypothesized that IFX good responders are more likely to have a TNF-α-driven disease, leading to a lower BMI. Nonetheless, caution should be taken with the hypothesis about a direct role of fat tissue-related mediators in AS, as to date, this remains speculative, and robust data are lacking.

Taking into account the increasing number of obese patients worldwide, investigation of the consequences of an excess of fat mass on drug metabolism is not trivial. Further studies have shown a direct influence of obesity on drug pharmacokinetics, notably regarding chemotherapy [24] and antihypertensive drugs [25,26]. Consequently, clearly a need exists for further work in elucidating the complex mechanisms involved in the development of obesity-associated resistance to IFX.

This study has some limitations. The design of our study was retrospective, which can lead to different biases, notably, the lack of pharmacokinetics analyses including correlation study between the IFX clearance and BMI. Additionally, we did not have detailed data on concurrent drugs that could be related to the overweight/obese status, and, therefore, are unable to assess the impact of the therapies on the results. However, regarding the BMI, similar findings were recently reported in a study in RA, and preliminary prospective data, not yet published, support our conclusions [27]. BMI is an indirect measure of body composition. Hence, a direct quantification of the body fat mass and fat-free mass should be assessed by using skinfold measurement, bioelectrical impedance analysis, or dual-energy x-ray absorptiometry to understand better the effect of each tissue in pharmacokinetics and adipocytokine production.

## Conclusion

In conclusion, with very recent data observed in RA, we provide the first evidence that a higher BMI negatively influences the response to IFX in AS. Further prospective studies are now required to determine how this must be taken into account for the treatment of RA and AS patients with IFX and probably other biologic agents. In addition, more data, including more-precise assessment of the fat mass, adipocytokines release, and pharmacokinetic study of the drug, are needed to elucidate the mechanism by which fat mass affects response to IFX in inflammatory rheumatic conditions.

## Abbreviations

AS: ankylosing spondylitis; BASDAI: Bath Ankylosing Spondylitis Disease Activity Index; BMI: body mass index; CRP: C-reactive protein; HLA: human leukocyte antigen; IFX: infliximab; NSAIDs: nonsteroidal antiinflammatory drugs; RA: rheumatoid arthritis; TNF: tumor necrosis factor; VAS: visual analogue scale; V_d_: volume of distribution; WHO: World Health Organization.

## Competing interests

The authors declare that they have no competing interests.

## Authors' contributions

SO and PD conceived of the study, made substantial contributions to the acquisition of data, participated in its design, and helped to draft the manuscript. FT and BP participated in the design of the study and performed the statistical analysis. MF, YA, AG, GH, MM, CJD, AK, and EP made substantial contributions to the acquisition of data. OM and YA helped to draft the manuscript. All authors read and approved the final manuscript.

## References

[B1] WHO Library Cataloging-in-Publication data Obesity: preventing and managing the global epidemic Technical Report Series 894 2000 http://www.who.int/nutrition/publications/obesity/WHO_TRS_894/en/11234459

[B2] Hanley MJ Abernethy DR Greenblatt DJ Effect of obesity on the pharmacokinetics of drugs in humans Clin Pharmacokinet 2010 49 71 87 10.2165/11318100-000000000-00000 20067334

[B3] Tilg H Moschen AR Adipocytokines: mediators linking adipose tissue, inflammation and immunity Nat Rev Immunol 2006 6 772 783 10.1038/nri1937 16998510

[B4] Muller-Ladner U Neumann E Rheumatoid arthritis: the multifaceted role of adiponectin in inflammatory joint disease Nat Rev Rheumatol 2009 5 659 660 10.1038/nrrheum.2009.232 19946293

[B5] Roubenoff R Roubenoff RA Ward LM Holland SM Hellmann DB Rheumatoid cachexia: depletion of lean body mass in rheumatoid arthritis: possible association with tumor necrosis factor J Rheumatol 1992 19 1505 1510 1464859

[B6] Briot K Gossec L Kolta S Dougados M Roux C Prospective assessment of body weight, body composition, and bone density changes in patients with spondyloarthropathy receiving anti-tumor necrosis factor-alpha treatment J Rheumatol 2008 35 855 861 18381782

[B7] Klaasen R Wijbrandts CA Gerlag DM Tak PP Body mass index and clinical response to infliximab in rheumatoid arthritis Arthritis Rheum 2011 63 359 364 10.1002/art.30136 21279992

[B8] Dougados M van der Linden S Juhlin R Huitfeldt B Amor B Calin A Cats A Dijkmans B Olivieri I Pasero G Veys E Zeidler H The European Spondylarthropathy Study Group preliminary criteria for the classification of spondylarthropathy Arthritis Rheum 1991 34 1218 1227 10.1002/art.1780341003 1930310

[B9] Braun J Pham T Sieper J Davis J van der Linden S Dougados M van der Heijde D International ASAS consensus statement for the use of anti-tumour necrosis factor agents in patients with ankylosing spondylitis Ann Rheum Dis 2003 62 817 824 10.1136/ard.62.9.817 12922952 PMC1754665

[B10] Vastesaeger N van der Heijde D Inman RD Wang Y Deodhar A Hsu B Rahman MU Dijkmans B Geusens P Vander Cruyssen B Collantes E Sieper J Braun J Predicting the outcome of ankylosing spondylitis therapy Ann Rheum Dis 2011 70 973 981 10.1136/ard.2010.147744 21402563 PMC3086037

[B11] Lord PA Farragher TM Lunt M Watson KD Symmons DP Hyrich KL Predictors of response to anti-TNF therapy in ankylosing spondylitis: results from the British Society for Rheumatology Biologics Register Rheumatology (Oxford) 2010 49 563 570 10.1093/rheumatology/kep422 20032223 PMC2820265

[B12] Lindegard B Diseases associated with psoriasis in a general population of 159,200 middle-aged, urban, native Swedes Dermatologica 1986 172 298 304 10.1159/000249365 3089849

[B13] Naldi L Addis A Chimenti S Giannetti A Picardo M Tomino C Maccarone M Chatenoud L Bertuccio P Caggese E Cuscito R Impact of body mass index and obesity on clinical response to systemic treatment for psoriasis: evidence from the Psocare project Dermatology 2008 217 365 373 10.1159/000156599 18810241

[B14] Puig L Obesity and psoriasis: body weight and body mass index influence the response to biological treatment J Eur Acad Dermatol Venereol 2011 25 1007 1011 10.1111/j.1468-3083.2011.04065.x 21492252

[B15] Saraceno R Schipani C Mazzotta A Esposito M Di Renzo L De Lorenzo A Chimenti S Effect of anti-tumor necrosis factor-alpha therapies on body mass index in patients with psoriasis Pharmacol Res 2008 57 290 295 10.1016/j.phrs.2008.02.006 18400510

[B16] Wiese D Lashner B Seidner D Measurement of nutrition status in Crohn's disease patients receiving infliximab therapy Nutr Clin Pract 2008 23 551 556 10.1177/0884533608323421 18849561 PMC4447197

[B17] Nakahigashi M Yamamoto T Increases in body mass index during infliximab therapy in patients with Crohn's disease: an open label prospective study Cytokine 2011 56 531 535 10.1016/j.cyto.2011.07.013 21820319

[B18] Rudwaleit M Listing J Brandt J Braun J Sieper J Prediction of a major clinical response (BASDAI 50) to tumour necrosis factor alpha blockers in ankylosing spondylitis Ann Rheum Dis 2004 63 665 670 10.1136/ard.2003.016386 15037444 PMC1755042

[B19] van der Heijde D Dijkmans B Geusens P Sieper J DeWoody K Williamson P Braun J Efficacy and safety of infliximab in patients with ankylosing spondylitis: results of a randomized, placebo-controlled trial (ASSERT) Arthritis Rheum 2005 52 582 591 10.1002/art.20852 15692973

[B20] St Clair EW Wagner CL Fasanmade AA Wang B Schaible T Kavanaugh A Keystone EC The relationship of serum infliximab concentrations to clinical improvement in rheumatoid arthritis: results from ATTRACT, a multicenter, randomized, double-blind, placebo-controlled trial Arthritis Rheum 2002 46 1451 1459 10.1002/art.10302 12115174

[B21] Krzysiek R Breban M Ravaud P Prejean MV Wijdenes J Roy C Henry YD Barbey C Trappe G Dougados M Emilie D Circulating concentration of infliximab and response to treatment in ankylosing spondylitis: results from a randomized control study Arthritis Rheum 2009 61 569 576 10.1002/art.24275 19405015

[B22] Villela NR Kramer-Aguiar LG Bottino DA Wiernsperger N Bouskela E Metabolic disturbances linked to obesity: the role of impaired tissue perfusion Arq Bras Endocrinol Metab 2009 53 238 245 10.1590/s0004-27302009000200015 19466216

[B23] Derdemezis CS Filippatos TD Voulgari PV Tselepis AD Drosos AA Kiortsis DN Effects of a 6-month infliximab treatment on plasma levels of leptin and adiponectin in patients with rheumatoid arthritis Fund Clin Pharmacol 2009 23 595 600 10.1111/j.1472-8206.2009.00717.x 19563510

[B24] Miya T Goya T Fujii H Ohtsu T Itoh K Igarashi T Minami H Sasaki Y Factors affecting the pharmacokinetics of CPT-11: the body mass index, age and sex are independent predictors of pharmacokinetic parameters of CPT-11 Invest New Drugs 2001 19 61 67 10.1023/A:1006456717846 11291833

[B25] Sharma AM Pischon T Engeli S Scholze J Choice of drug treatment for obesity-related hypertension: where is the evidence? J Hypertens 2001 19 667 674 10.1097/00004872-200104000-00001 11330867

[B26] Cortinez LI Anderson BJ Penna A Olivares L Munoz HR Holford NH Struys MM Sepulveda P Influence of obesity on propofol pharmacokinetics: derivation of a pharmacokinetic model Br J Anaesth 2010 105 448 456 10.1093/bja/aeq195 20710020

[B27] Ferraccioli G Trotta F Punzi L Ferri C Sarzi-Puttini P Bambara L Triolo G Giacomelli R Gerli R Gorla R Marchesoni A Grassi W Lapadula G Weight and response to biologics in RA and spondylarthritides: obesity reduces the rate of remission-response: The GISEA Registry [Abstract] Arthritis Rheum 2010 62 297

